# Tissues and Cell Types of Appendage Regeneration: A Detailed Look at the Wound Epidermis and Its Specialized Forms

**DOI:** 10.3389/fphys.2021.771040

**Published:** 2021-11-23

**Authors:** Can Aztekin

**Affiliations:** Swiss Federal Institute of Technology Lausanne, EPFL, School of Life Sciences, Lausanne, Switzerland

**Keywords:** wound epidermis, specialized wound epidermis, AEC, single-cell transcriptomics, appendage regeneration, limb regeneration

## Abstract

Therapeutic implementation of human limb regeneration is a daring aim. Studying species that can regrow their lost appendages provides clues on how such a feat can be achieved in mammals. One of the unique features of regeneration-competent species lies in their ability to seal the amputation plane with a scar-free wound epithelium. Subsequently, this wound epithelium advances and becomes a specialized wound epidermis (WE) which is hypothesized to be the essential component of regenerative success. Recently, the WE and specialized WE terminologies have been used interchangeably. However, these tissues were historically separated, and contemporary limb regeneration studies have provided critical new information which allows us to distinguish them. Here, I will summarize tissue-level observations and recently identified cell types of WE and their specialized forms in different regeneration models.

## Introduction

Limb regeneration is associated with two heterogeneous tissue types: a specialized wound epithelium that caps the amputation plane, and a blastema which forms underneath the specialized wound epidermis (WE) and contains lineage-restricted stem and progenitor cells that will give rise to the new appendage (Beck et al., 2009; Aztekin, 2021). The interaction between these two tissues leads to the outgrowth of the lost structure. Studies with newts, which can consistently perform limb regeneration throughout their lifespan, provided landmark findings governing the function of these tissues (Joven et al., 2019). Subsequent reports aimed to characterize and identify counterparts in other regeneration models.

The critical role of the WE for regenerative success stems from the observations dating back to the early 1900s (Morosow, 1938; Thornton, 1960). Mainly, newt limb amputations were found to progress with a rapid epithelial migration resulting in the closure of the amputation plane (Lash, 1955; Hay and Fischman, 1961; Repesh and Oberpriller, 1978), forming the WE. Afterward, the WE progresses into its specialized morphologically thickened epithelial form, also known as the apical epithelial cap (AEC). Repeated salamander AEC removal or blocking AEC formation halts the limb regeneration program, highlighting its essential role for limb regeneration (Morosow, 1938; Goss, 1956; Thornton, 1957, 1958; Mescher, 1976; Tassava and Garling, 1979; Tsai et al., 2020). Conversely, grafting the salamander AEC can induce ectopic limb outgrowths, with cartilage and dermal composition (Thornton, 1960; Thornton and Thornton, 1965). Owing to these features, the derivation of the AEC for grafting or identifying genes associated with the AEC holds potential for therapeutic applications. However, before delving into the properties of the WE and the AEC, it is crucial to define them clearly.

**FIGURE 1:**
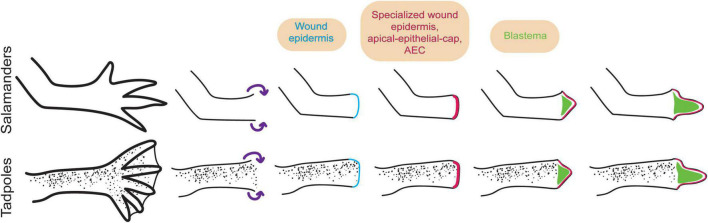
The wound epidermis (WE) and the specialized wound epidermis form in a step-wise manner during amphibian limb regeneration. Limb regeneration is initiated with amputation in (top) salamanders and (bottom) tadpoles. The remaining stump epidermal cells migrate to the amputation plane (purple arrows) and form the WE (light blue). Then the WE becomes the specialized wound epidermis [apical-epithelial-cap (AEC)] (dark pink) associated with regeneration (Christensen and Tassava, 2000). Afterward, the AEC leads to blastema formation and subsequent outgrowth (Tassava and Loyd, 1977). Although the presence of the AEC in early and mid-stages of regeneration is demonstrated by tissue morphology and staining assessments (e.g., Han et al., 2001), its presence in late stages is observed only with tissue morphology assessment.

The term “AEC” was initially used to identify an epithelium covering aggregated blastema cells during limb regeneration. Nonetheless, this definition could be used for both the WE and the AEC. Moreover, investigations on different non-limb regeneration scenarios (e.g., tail regeneration) sought to identify similar tissues. However, due to unclear definitions of these tissues, the WE and the AEC terminologies were used interchangeably. To distinguish them and clarify their differences, Christensen and Tassava (2000) suggested a revision to these terminologies. Briefly, the WE is the epithelium covering the amputation plane (right after amputations), remains on the amputation plane for a short period, and has a simple morphology consisting of one to three layers of cells (Figure 1). Meanwhile, its specialized form, the AEC, appears after the WE formation. The AEC remains until proximal-distal elongation (until digit formation), and has a thickened structure of approximately 10–15 layers of cells (Figure 1). On this basis, two features were proposed to distinguish them: (1) the period coinciding with their presence during regeneration and (2) tissue morphology. Although these definitions are helpful, our current methodologies evidenced that tissue or cellular morphology could be inadequate assessments for functionally distinct populations and cell types. Moreover, the different cell types composing and identifying the WE or the AEC were not resolved. Instead, investigating their counterparts in other species or regeneration paradigms brought additional misperception to these concepts.

In this review, I aim to bridge historical tissue level observations of the WE and the AEC with recent single-cell transcriptomics and other cell-type focused findings, primarily in the context of vertebrate limb regeneration. Due to the interchangeable documentation of these two concepts, I will focus on the properties of the AEC and mark potential phenotypes distinguishing it from the WE. Finally, I will compare the cross-species and regeneration-model properties of the WE and the AEC.

## What Does the Apical-Epithelial-Cap, Do?

The salamander AEC has been associated with multiple critical roles for successful regeneration, from influencing extracellular-matrix (ECM) organization to providing mitogenic factors. Among these undertakings, one of the early suggested AEC functions governed the question: does the blastema influence the AEC formation or vice versa? Work targeting this question proposed that the injury caused by the amputation induces morphologically identified dedifferentiated cells (Tassava and Loyd, 1977). These cells can proliferate and form a blastema in the presence of the AEC. However, without the AEC, the blastema does not form, although dedifferentiated cells can be observed. Hence, the AEC has been suggested to maintain and further instruct injury-induced dedifferentiated cells to form a blastema and patterning for regeneration. Due to the observation that injury-induced dedifferentiated cells can form without the AEC, it has been hypothesized that the lack of a specialized WE results in regeneration-incompetency in higher vertebrates (Tassava and Olsen, 1982).

In subsequent years, the AEC has been further associated with multiple essential cellular mechanisms for regenerative success. The majority of functional suggestions on the role of the AEC were concluded based on staining approaches and observed gene expression patterns. For example, the salamander AEC expresses ECM associated *fn1* (Christensen and Tassava, 2000), *collagen type XII* (Wei et al., 1995), *collagen type IV, lamb1* (Del Rio-Tsonis et al., 1992), *krt5*, *krt17* (Moriyasu et al., 2012), and *frem2* (Leigh et al., 2018); histolysis involved *mmp3/10b* and *mmp9* (Yang et al., 1999; Vinarsky et al., 2005); mitogenic factors such as *fgf1* (Boilly et al., 1991), *fgf2* (Mullen et al., 1996), *fgf8* (Han et al., 2001; Christensen et al., 2002), *fgf10* (Christensen et al., 2002), *mdk* (Tsai et al., 2020), and MARCKS-like (Sugiura et al., 2016); angiogenesis-related *tsp1* (Whited et al., 2011); and chemotactic factors as *wnt5a* (Ghosh et al., 2008). The existence of multiple AEC derived factors could either enable functional redundancy or allow for cell-type-specific interpretations. For example, some of these factors would influence muscle and some others would influence connective tissue lineages. Furthermore, several transcription factors are associated with the salamander AEC: *tp63* (Kawakami et al., 2006), *msx2* (Carlson et al., 1998), *dlx3* (Beauchemin and Savard, 1992), *id2*, *id3*, *hes1* (Shimizu-Nishikawa et al., 1999), and *sp9* (Satoh et al., 2008), although further work is required to reveal their molecular role during AEC establishment or maintenance. The function of the AEC and its formation have been investigated by systemic perturbations rather than tissue or cell type-specific functional assessments. As an example, the AEC is documented to express various metalloproteinases (MMP) associated with histolysis that degrade ECM components critical for regenerative success, and MMP inhibitors can inhibit limb regeneration (Vinarsky et al., 2005). Nonetheless, it remains unclear if and how much the AEC-derived MMPs result in histolysis that could be required for regeneration. Bulk sequencing approaches to the whole AEC tissue were employed and expanded potential genes related to the AEC and identified new functionally critical gene targets for regeneration, such as *mdk* (Monaghan et al., 2012; Knapp et al., 2013; Tsai et al., 2019, 2020). Due to methodological limitations, bulk approaches alone could not discriminate which critical cell types within the AEC tissue are responsible for these gene expressions or if the whole AEC tissue expresses them. Overall, as the AEC has been primarily associated with secreted ligands, it is largely regarded as a signaling center orchestrating cellular mechanisms that are vital for successful regeneration.

## Is the Apical Epithelial Cap Formation Re-Deployment of the Limb Development Associated Apical-Ectodermal Ridge?

One of the main questions in regeneration biology involves revealing similarities between regeneration and development. From this perspective, the AEC has been associated with the apical ectodermal ridge (AER), which is well-established transient tissue essential for chicken and mouse limb development (Fernandez-Teran and Ros, 2008). Like the AEC, the AER also represents a heterogeneous tissue forming at the distal tips of limb buds; the AER is also covered with a periderm and marked with *fgf8* at the basal layers (Nakamura and Yasuda, 1979; Aztekin et al., 2021). Moreover, multiple classical AER genes (e.g., *fgf2*, *fgf8*, *sp9*, and *fn1*) are also expressed in the AEC. The absence of the chicken AER or mutations impairing the mouse AER results in no limb development (Saunders, 1948; Fernandez-Teran and Ros, 2008), and the lack of the salamander AEC results in no limb regeneration (Goss, 1956; Thornton, 1957, 1958; Mescher, 1976; Tassava and Garling, 1979). Akin to the amphibian AEC, the amniotic AER was suggested to maintain and enable the self-renewal of underlying limb progenitors (Tassava and Mescher, 1975; Mescher, 1976; Tassava and Loyd, 1977). Although not studied in detail, the presence of the AER was suggested to influence angiogenesis (Tschumi, 1957) and muscle-cell migration for limb development (Gumpel-Pinot et al., 1984; Francis-West et al., 2003). Moreover, the AER was proposed to influence specific limb progenitor cells to express early limb progenitor markers (Watanabe et al., 1993; Mariani et al., 2008), highlighting AER-derived signals may influence progenitor cell states, although this feature has been debated (Pickering et al., 2018; McQueen and Towers, 2020).

Both the AER and the AEC were suggested to be largely mitotically inactive populations (Moriyasu et al., 2012; Storer et al., 2013; Aztekin et al., 2021), and their formation requires the activity of the well-studied signaling pathways (e.g., FGF, BMP, and WNT; Fernandez-Teran and Ros, 2008). Notably, both the AEC and the AER require signals, such as *Fgf10*, from their underlying mesodermal cells to form and maintain themselves (Ohuchi et al., 1997, 1999; Yokoyama et al., 2001). Ectopic application of FGF10, or B-catenin overexpression can induce both AEC and AER formation in different species (e.g., chicken and *Xenopus*; Yonei-Tamura et al., 1999; Kawakami et al., 2001, 2006; Yokoyama et al., 2001; McQueeney et al., 2002). Although they have many similarities, the AEC and the AER could have distinct transcriptomic signatures making the AEC a novel population with new functions forming during regeneration. Moreover, revealing the similarity between the AER and the AEC also provides new insight for our understanding of human limb regeneration. As the AER forms during human embryonic development (Kelley and Fallon, 1976), human epithelial cells may have the competency to form the AEC, and mammalian basal epidermal cells could be used to derive functional AER cells. Hence, it is critical to determine if the AEC represents a novel population or re-usage of developmental programs.

Despite all similarities, the AEC and the AER are argued to have some differences, and there are challenges to testing their equivalency. First, morphological features of the AER and the AEC were discussed as potential differences (Campbell and Crews, 2008). While the AER can be found as a stratified ridge structure in mice and chicken (Fernandez-Teran and Ros, 2008), the AEC is mainly associated with a flat epidermal tissue (Campbell and Crews, 2008; Satoh et al., 2008). Moreover, unlike the AER, the AEC is observed to be a 10- to 15-cell-thick tissue (Campbell and Crews, 2008). Hence, variations in tissue morphology do not provide a new understanding of the functional differences between these two tissues. Second, the lineage relations and cellular mechanisms enabling the formation of the AER and the AEC are still debated. Both the AER and the AEC formation have been suggested to be mediated through a boundary model where dorsal and ventral epidermal populations interact to give rise to these tissues (Fernandez-Teran and Ros, 2008; Shimokawa et al., 2012). However, numerous phenotypes cannot be explained *via* the boundary model for the mouse AER (discussed in Fernandez-Teran and Ros, 2008). Moreover, it remains unclear which specific cellular and molecular mechanisms and lineage-relations guide AER or AEC formation. Although several secreted factors are identified to be critical and sufficient for AER formation from specific ectodermal populations (e.g., *Wnt pathway activity*, *Fgf10*, and *Fgf7;* Ohuchi et al., 1997; Yonei-Tamura et al., 1999; Yokoyama et al., 2001; McQueeney et al., 2002), it remains unclear how AER cell identity is established during development. Likewise, the molecular basis for AEC induction is currently not resolved. One possibility is that the amputation plane connective tissue lineage, the WE, or both may be secreting AER inducing signals to induce an AEC.

There are different models for AER disappearance during amniotic development (Scherz et al., 2004; Verheyden and Sun, 2008; Storer et al., 2013; Pickering et al., 2018), but how the AEC disappears during regeneration remains unknown. Salamander and froglet AEC formations have been suggested to be a nerve-dependent process (Suzuki et al., 2005; Satoh et al., 2008; Stocum, 2019), while nerves are not required for the *Xenopus* tadpole limb regeneration (Cannata et al., 1992). Furthermore, limb explants (that are presumably devoid of nerves) can still form the AEC (Aztekin et al., 2021). Meanwhile, the nerve dependence of the AER in amniotes is not suggested. Third, critically, the AER is separated from the underlying mesoderm by a basement membrane (Saunders, 1948) whereas, the newly formed AEC and underlying cells are in direct contact (Repesh and Oberpriller, 1978; Neufeld et al., 1996), presumably enabling easier interaction between these tissues. Fourth, the AEC is mostly characterized in salamanders which are suggested not to have an AER (Tank et al., 1977; Stopper and Wagner, 2005; Purushothaman et al., 2019), and the AER is mostly characterized in amniotes, which are suggested not to have the AEC. Hence, although the AER and the AEC share many features, their detailed characterization was not done in the same animal. Nonetheless, other limb regeneration-competent species could overcome some of these issues and may enable the determination of possible similarities between the AER and the AEC.

*Xenopus laevis* is the only commonly used laboratory animal with a well-established AER and AEC (Stopper and Wagner, 2005; Purushothaman et al., 2019). Using single-cell transcriptomics, we revealed individual cell types defining these tissues and compared their similarities at the single-cell transcriptome level (Aztekin et al., 2021). Instead of the AER and the AEC harboring different populations, individual cells found at the basal layers of these tissues share a similar transcriptome and appear in the same computational cluster. Nevertheless, the AEC has a higher potential as a signaling center, given that the same ligands show increased expression in the AEC compared to the AER. For example, although *fgf8* or *fn1* are expressed in the AER, their expression is significantly higher in the AEC, presumably creating a niche with very high levels of total ligands compared to the limb development environment. Further assessments on basal *fgf8* + epidermal cells during regeneration and development revealed a shared single-cell morphology, spatial organization, and common regulatory cellular mechanisms. During development and regeneration, basal epidermal cells in the AER and the AEC are seen to form from a stepwise activation of *lgr5* and then *fgf8* in basal epidermal cells in a spatially restricted manner, from proximal to the distal tip of a limb. However, the molecular mechanism enabling this differentiation remains unclear. Due to this very high degree of similarity, we proposed using the term “AER cells” for cells in the basal epidermal *fgf8* + cells formed during limb development and regeneration. On this basis, the AEC tissue contains cells harboring the AER cell transcriptional program at its basal epidermal layer. Although a functional equivalence assay will be essential, single-cell comparison and current functional assays represent a stringent evaluation supporting the hypothesis that the AEC is re-deployment of the AER, at least in *Xenopus*.

## Re-Assessing Morphological and Gene Expression Markers of the Apical Epithelial Cap

The salamander WE and the AEC were historically distinguished mainly based on their tissue morphology. Notably, the thickened epithelium was used to detect the AEC. Initial attempts aimed at identifying the AEC used antigen stainings (Tassava et al., 1986, 1993; Goldhamer et al., 1989; Tassava and Acton, 1989; Castilla and Tassava, 1992). These staining approaches also revealed that such antigens were enriched in secretory cells in the animal body, suggesting that the AEC might have a secretory cell phenotype (Goldhamer et al., 1989). The use of mRNA staining, particularly *fn1* (Christensen and Tassava, 2000) and *fgf8* (Han et al., 2001), suggested that the AEC could be a heterogeneous tissue and proposed that the basal layers may be the critical population for regeneration. AEC-associated genes are expressed at the basal layers of the AEC tissue 2–3 days after amputations but not necessarily in the middle or apical layers. These gene expressions are not found directly after amputations. Hence, cells defining the AEC are not identical to those in the WE, although there could be shared gene expressions [e.g., MARCKS-like (Sugiura et al., 2016) and *tsp1* (Whited et al., 2011)] and these two tissues may have lineage relations. Moreover, as the AEC-associated genes are mainly observed at the basal layers, these results were also suggesting thickened epithelial-morphology may not be providing AEC-associated regeneration promoting functions. Overall, not all cell types and transcriptional programs are identical within the AEC itself or between the AEC and the WE.

Marker genes distinguishing the AEC from the WE have been revealed. Particularly, *fgf8* has been associated with the salamander limb AEC, but not the WE (Han et al., 2001; Christensen et al., 2002). However, recent years provided inconsistent results for *fgf8* expression in axolotl limb regeneration. Initial characterizations suggested *fgf8* marks a population in the basal layers of the AEC (Han et al., 2001; Christensen et al., 2002). In the accessory limb model where a secondary limb is induced from an upper arm *via* specific experimental perturbation, *fgf8* expression was not found (Nacu et al., 2016). Later work suggested that *fgf8* expression is not restricted to the basal level layers (Vincent et al., 2020). Although the validity of *fgf8* to detect the axolotl AEC presents certain caveats, new potential AEC markers were shown to label basal layers of the AEC [e.g., *mdk* (Tsai et al., 2020), *wnt5a* (Ghosh et al., 2008)] and are not expressed in the WE, in the initial days postamputation. While *mdk* and *wnt5a* expression can be observed in other cell populations, this gene expression pattern in basal epidermal cells labels the AEC. Overall, it remains unclear if *fgf8* is expressed during axolotl limb regeneration, but there are multiple alternative markers to detect the AEC in the salamanders.

*Fgf8* expressing basal epidermal cells in the AEC have been characterized during *X. laevis* limb regeneration (Christen and Slack, 1997; Wang and Beck, 2014; Aztekin et al., 2021). Unlike salamanders, *X. laevis* can regrow their lost limb in early limb bud stages but lose this ability progressively during their development (Dent, 1962). When tadpole limbs are in their secondary growth, they can only regrow a limb with a few digits. Afterward, amputations produce one out of two phenotypes toward metamorphosis: either simple wound healing or spike formation. Simple wound-healing phenotype shows epidermal covering of the amputation plane and no growth, meanwhile, the spike formation involves the growth of an unpatterned cartilaginous rod that lacks muscles and bones (Satoh et al., 2005). During regeneration-competent stages, the *Xenopus* AEC has been reported to contain abundant *fgf8* expressing cuboidal cells in basal layers (Pearl et al., 2008; Aztekin et al., 2021). Meanwhile, when tadpoles progressively lose their limb regeneration competency, they also produce fewer *fgf8* + cells in the AEC, and when they are regeneration-incompetent, there are no *fgf8* + cells at the amputation plane; although these animals can still seal their amputation plane, therefore can form the WE (Christen and Slack, 1997; Aztekin et al., 2021). Unlike regeneration-incompetent tadpoles, amputated postmetamorphic froglets homogenously perform spike formation but not simple wound healing (Beck et al., 2009). There are different results for where and when the *fgf8* is expressed for postmetamorphic *Xenopus* froglets that can consistently regrow a spike but not perform simple wound healing, for where and when the *fgf8* is expressed (Endo et al., 2000; Suzuki et al., 2005; Satoh et al., 2017). Moreover, it remains unclear if the formed froglet *fgf8* population is equivalent to ones in regeneration-competent limbs.

## Single-Cell Studies Reveal Cell Types Defining Limb-Specific Apical Epithelial Cap

With recent advancements in cell-centric approaches, we now have an opportunity to characterize specific cell types within the WE and the AEC tissues. Although definitions of a cell type or its transient forms as a cell state have been debated (Trapnell, 2015; Arendt et al., 2016; McKinley et al., 2020), single-cell RNA sequencing (scRNA-Seq) approaches reveal transcriptome profiles for individual cells that can suggest specific functions. Moreover, with scRNA-Seq, we can now obtain more extensive gene expression profiles, expanding and clarifying the AEC-associated gene lists. Cells with highly similar transcriptomes form computationally assigned clusters, representing potential cell types or states. As in every computational method, analysis can yield artificial scenarios. Over/underclustering or analysis with low-quality or non-comprehensive datasets can suggest cell types or states that are not necessarily reflecting functionally distinct populations. Although cell types harbor stable gene regulatory networks associated with their identity and established functions, scRNA-Seq records the transcriptomes of a single time point, while cells are dynamic and change their transcriptome constantly. Moreover, scRNA-Seq can only capture transcriptional information meanwhile there could be posttranscriptional responses determining cellular functions. Therefore, it is critical to test the quality and validity of single-cell atlases, and more critically, validate the computational suggestions with experimental approaches. Secondly, contrary to traditional non-quantitative staining methods (e.g., antibody labeling, colorimetric *in situ* hybridization), single-cell transcriptomics and new mRNA staining approaches offer a more unbiased and quantitative methodology to discriminate lowly or highly expressed genes, which can again be an indication for specific cellular functions. These advancements allow us to identify and detect cell types *in situ*. On this basis, we can uncover cell types representing WE and AEC tissues and ask if they could be found in different species and regeneration paradigms.

From the initial studies in salamanders, it is well established that the salamander WE will form *via* the migration of remaining stump epidermal cells (Hay and Fischman, 1961). Hence, cells composing the WE can be expected to be already present before amputation in scRNA-Seq datasets as a basal epidermal cell cluster in homeostatic states of limbs. Meanwhile, based on the literature, the AEC could be reflected in these single-cell maps as a new cluster that forms during regeneration and expresses marker genes such as *fn1*, *fgf8*, *wnt5a*, and *mdk*, in addition to having a basal epidermal cell signature (e.g., *tp63*). Based on these criteria, the new advances in single-cell analysis provide novel insights into the cellular properties of the WE and the AEC tissues.

Several axolotl limb regeneration studies incorporated scRNA-Seq (Gerber et al., 2018; Leigh et al., 2018; Li et al., 2020; Qin et al., 2020; Rodgers et al., 2020), with some targeting the whole newly forming cells, including epidermal populations. In one of these studies, Leigh et al. detected basal epidermal clusters and validated *frem2* as a new marker (Leigh et al., 2018). In this study, this *frem2* expressing population was also found in the limb homeostatic state and was not suggested as an emerging population upon amputation. Qin et al. (2020) identified a separate basal epidermal population expressing known AEC markers (e.g., *mdk* and *fn)* and suggested they represent the AEC. Contrary to previous studies, this population is also seen before amputation. Hence, it remains unclear whether AEC cells are present in the axolotl before amputation or if alternatively, these studies identified a population representing a mix of basal epidermal cells due to the experimental design and/or analysis. Interestingly, these studies did not report a separate *fgf8* + basal epidermal population. Further investigation on these datasets and validatory experiments will be required to pinpoint the WE and the AEC in the axolotl limb regeneration.

Single-cell investigation on *Xenopus* limb regeneration, focusing on the AEC, confirmed that the AEC is a heterogeneous tissue and that only the basal layers contain *fgf8* + cells (Aztekin et al., 2021). Moreover, scRNA-Seq revealed that not all *fgf8* + epidermal cells are equivalent. Intriguingly, the level of *fgf8* expression correlates with the signaling center potential of basal epidermal cells; if *fgf8* expression is high, the expression of other ligands (Fgf, Bmp, Wnt, Tgfb, and Delta) involved in major signaling pathways is also high. Moreover, *Xenopus* AER cells express lower levels of *fgf8* in regeneration-restricted tadpoles (which can only grow back a foot with two to three digits) compared to regeneration-competent AER cells. Hence, not just the expression of *fgf8*, but its *expression level* is a new parameter to consider while determining the properties of the AEC and may indicate potentially different generated morphogen gradients generated for limb regeneration. From a technical point of view, these findings imply that caution is necessary when detecting the AEC. Methodologies that cannot reliably discriminate low and high gene expressions may not determine the signaling center potency of the formed AEC, which is indicative of how many ligands are expressed. Therefore, detection of basal epidermal *fgf8* + cells relying more consistently on more user-unbiased and quantitative mRNA staining approaches [e.g., hybridization-chain-reaction (Choi et al., 2018), single amplification by exchange reaction fluorescence *in situ* hybridization (Kishi et al., 2019), scRNA-Seq] will be essential to study properties of the AEC. Moreover, some of the key genes detected in the salamander AEC are conserved for the *Xenopus* AEC (e.g., *fgf8*, *fn1*, *mdk*, and *wnt5a*). However, there are also some differences, such as, *sp9* which is expressed in the salamander AEC, but its expression is not significantly detected in the *Xenopus* AEC (Aztekin et al., 2021). Further cellular comparisons between species can highlight conserved features of the AEC.

## Divergent Molecular, Cellular, and Tissue-Level Properties of Wound Epidermis and Apical Epithelial Cap in Other Regeneration Models

The WE and the AEC have been long sought and investigated in other regeneration models. Currently, the epidermal population covering the amputation plane in different regeneration models is also named the WE or the AEC. However, due to different tissue formation kinetics in other species and regeneration paradigms, differences between two tissues are even less precisely defined than those in amphibians.

**FIGURE 2:**
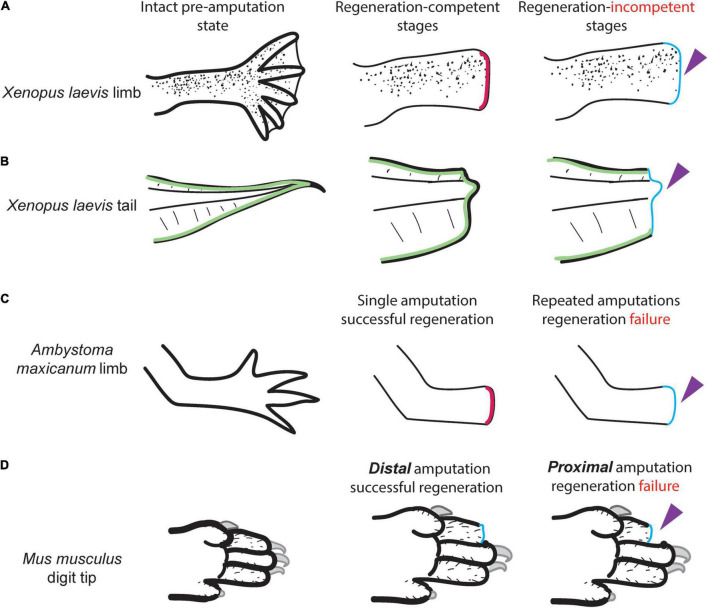
The wound epidermis (WE) formation is not associated with regenerative success in diverse appendage regeneration models. **(A, B)** *Xenopus laevis* tadpoles lose their limb and tail regeneration abilities at specific developmental stages. Upon amputations, these animals cannot form an AEC (dark pink) at these stages but can still form the WE (light blue; Beck et al., 2009). **(A)** *X. laevis* tadpole limb regeneration ability is associated with the successful specification of AER cells to form the AEC; regeneration-incompetent tadpoles cannot specify AER cells but can still seal the amputation plane, hence they can form the WE (Aztekin et al., 2021). **(B)** *X. laevis* tail regeneration depends on the ability to relocalize their regeneration-organizing cells (ROCs; green) to amputation plane to act as the AEC (Aztekin et al., 2019). Regeneration-incompetent tadpoles cannot relocalize their ROCs, but can still seal the amputation plane, hence they can form the WE. **(C)** Repeated amputation of the axolotl limb results in no regeneration that can form a WE (Bryant et al., 2017b). **(D)** Mouse digit tip regeneration is amputation position-dependent, yet both distal regenerative and proximal regeneration deficient amputations result in the WE formation (Simkin et al., 2015).

Recent research with regeneration models that show both regeneration-competency and -incompetency further stresses the need for re-evaluation to distinguish the WE and the AEC terminologies. In heavily studied appendage regeneration competent vertebrate species, the amputation plane is sealed with a simple epithelium that fits the WE definition. However, this ability can be observed even in regeneration-competent species that are exhibiting regeneration-incompetency. For example, limb amputation in regeneration-incompetent *Xenopus* tadpoles can result in simple wound healing and can still display the WE formation (Dent, 1962; Figure 2A). Likewise, *X. laevis* can regenerate its lost tail before metamorphosis except for a brief period during its development (Niewkoop & Faber Stage 46–47), referred to as the refractory period (Beck et al., 2003). During this period, amputated tails do not regenerate; however, the amputation plane is still covered with an epidermis, hence tadpoles can form the WE (Beck et al., 2003; Aztekin et al., 2019; Figure 2B). These results suggest that the WE may not have a functional role for *Xenopus* tail or limb regeneration, at least for regeneration purposes. Repeated amputations of the axolotl limbs result in no regeneration, yet a wound epithelium still forms (Bryant et al., 2017a; Figure 2C). In addition, in this setting, the formed WE exhibit a thickened morphology distinct from the AEC associated thickness (Bryant et al., 2017b), and it remains unclear if repeated amputations can form the AEC.

Mice can regrow their distal digit tip throughout their life, and distal digit tip regeneration undergoes the WE formation, although this process takes much longer than amphibian limb and tail regeneration scenarios. By contrast, amputating more proximal digit tips results in no regeneration, yet an epithelium, which can be regarded as the WE, still covers the amputation plane (Dawson et al., 2021; Figure 2D). In contrast to *Xenopus* tail regeneration, the mouse digit tip WE seem to have a functional role in tissue histolysis by boosting the blastema formation (Simkin et al., 2015). In another appendage regeneration model, deers can regrow their lost antlers, in which the WE forms but does not necessarily influence the regrowth of the antler (Li et al., 2007, 2014). Hence, WE formation seems to be conserved across regeneration-competent species, although it does not correlate with successful appendage regeneration. Beyond these differences, tail regeneration in axolotl, zebrafish, or *Xenopus*, or mouse digit tip does not produce a thickened epithelium that shows morphological similarity to the amphibian limb regeneration associated AEC (Beck et al., 2003; Lehoczky et al., 2011; Pfefferli and Jaźwińska, 2015; Dolan et al., 2018). Altogether, neither the timing nor the tissue-morphology could be used to discriminate or label specific populations as the WE or the AEC across species, and their conserved role for regeneration remains ambiguous.

Gene expression profiles were tested to identify the WE and the AEC across species and regeneration paradigms, and some common gene expressions were found across certain species. For example, *lef1*, *tp63*, and *msx family* were expressed in the amputation plane during *Xenopus* tail and limb (Kawakami et al., 2006; Aztekin et al., 2019, 2021), lizard tail (Vitulo et al., 2017), and zebrafish caudal fin regeneration (Akimenko et al., 1995; Poss et al., 2000). However, these genes are not specifically seen in the WE or the AEC. Meanwhile, the canonical AEC marker *fgf8* is absent in zebrafish caudal fin epidermis covering the amputation plane (Shibata et al., 2016). Interestingly, zebrafish pectoral fin amputations were suggested to form the AER due to re-expression of basal epidermal *rspo2* after amputations, which is also used to detect *fgf8* expressing AER (Yoshida et al., 2020). For *X. laevis* tail regeneration, the expression pattern of *fgf8* is conflicted in different reports: while colorimetric *in situ* hybridizations detect *fgf8* in the AEC with inconsistent patterns (Beck et al., 2006; Lin and Slack, 2008; Okumura et al., 2019), bulk-RNA-Seq and scRNA-Seq based results suggest *fgf8* is not expressed in the *Xenopus* tail AEC (Aztekin et al., 2019; Okumura et al., 2019). Overall, currently, there is no identified specific pan-AEC marker gene found across species or regeneration paradigms.

Cross-regeneration model comparisons indicate that the AEC may not be a shared tissue type for appendage regeneration, and cell types defining the AEC may show differences. Indeed, by using bulk-RNA sequencing, *Xenopus* tail and limb AEC tissues are shown to exhibit gene expression differences (Okumura et al., 2019). Moving beyond tissue to the single-cell level, we identified that *Xenopus* tail regeneration uses an epithelial population resembling transcriptional programs similar to the AER (Aztekin et al., 2019). Due to their essential role during tail regeneration, AEC-associated gene expression, and high signaling center properties, we named this population regeneration-organizing cells (ROCs). The subsequent single-cell-based analysis demonstrated that AER cells and ROCs do not share an identical transcriptome nor single-cell morphology (Aztekin et al., 2021). Beyond transcriptomic differences, their formation on the amputation plane uses different cellular mechanisms, emphasizing that they function differently. AER cells have to be re-specified to form defining properties of the signaling center AEC for limb regeneration (Figure 2A). Meanwhile, the inability to form and maintain AER cells is observed in regeneration-incompetent tadpoles. During tail regeneration, ROCs are already present at the basal layers of midline epidermis before tail amputations, and their relocalization to the amputation plane provide the signaling center AEC for tail regeneration (Figure 2B). Meanwhile, this relocalization is not seen in regeneration-incompetent tadpoles. ROCs are found as multilayered flattened cells, while AER cells are seen as a cuboidal monolayer population (Aztekin et al., 2021). Nonetheless, they are both involved in the expression of many ligands and act as a signaling center, fitting with the acknowledged functional role of the AEC. Further cross-species single-cell transcriptomics studies and functional assessments will be required to pinpoint if there are more cell types with signaling center abilities in different species. To this aim, the detection of basal epidermal cells showing high ligand gene expressions upon amputations could be used as a valuable readout. Identifying such cell types in other species can reveal their conserved functions and potential gene regulatory networks mediating the signaling center abilities for appendage regeneration.

## Conclusion

The critical functions of the AEC and its requirement for blastema formation and limb regeneration are widely accepted. Since their initial discovery, definitions of the WE and the AEC and their detection methodologies have not reached consensus across species and regeneration models. Based on current literature and initial single-cell characterizations, basal epidermal populations that migrate and seal the amputation area could be considered the WE. These cells may exhibit many similarities among all regeneration-competent species. However, the presence of the WE does not correlate with regeneration success, and their cellular identities remain elusive. Meanwhile, the presence of AEC on the amputation plane is positively correlated with regenerative success, and basal epidermal cells that form after the WE and exhibit high signaling center properties could be a more suitable description for cell types defining the AEC. Nevertheless, cell types and critical genes defining the AEC do not show conservation across species and regeneration models. For example, ROCs relocalize to form the *Xenopus* tail AEC, meanwhile, AER cell formation for the *Xenopus* limb AEC involves a differentiation event. On this basis, extra caution is required while using these terminologies and searching for their counterparts in different regeneration models. There could be diverse cell types defining the AEC tissue (as exemplified by AER cells for *Xenopus* limbs and ROCs for *Xenopus* tails). Identifying if appendage regeneration can be mediated without such populations may reveal new mechanisms for appendage regeneration.

Beyond elucidating cell fates represented in the WE or the AEC tissues, the behavioral properties of such populations and the molecular mechanisms enabling their formation on the amputation plane remain largely unknown. For example, mechanical damage has been implicated in creating Erk activation that temporarily spreads from the damaged region to the surrounding area, forming wave patterns, influencing epithelial cell migration and survival (Aoki et al., 2017; Hino et al., 2020; Gagliardi et al., 2021). Yet the implication of such Erk activity spreading for wound closure and limb regeneration remains unknown. Likewise, amputations have been indicated to promote epithelial proliferation and involve forming an actomyosin cable during zebrafish fin regeneration (Mateus et al., 2012). Moreover, in zebrafish caudal fin and tail regeneration, a transient metabolic shift to glycolysis has been shown to influence actomyosin networks in epithelial cells for successful epithelial closure, presumably forming the WE (Scott et al., 2021). How such molecular processes and metabolic changes mediate rapid wound covering during limb regeneration remains unknown. In sum, identifying cell types, characterizing their cellular behaviors and molecular regulators can promote the development of novel approaches for mammalian WE and AEC formation.

Mammalian skin wound healing has been studied to uncover ways for scar-free healing and can provide perspectives for epithelial behaviors during limb regeneration. As an example, skin wound injuries have been shown to be covered with excessive proliferation of a select number of stem cell clones, rather than using mass migratory behaviors or cell-fate switches (Aragona et al., 2017). From this perspective, during amphibian limb regeneration, epithelial behaviors seem rather distinct compared to a mouse WE formation for simple injury. Identifying such cellular features in mammals and screening them during limb regeneration in amphibians can hint at potential links for regeneration-competency. For example, the amniotic epidermis is highly stratified and exhibits a higher degree of heterogeneity with its keratin-rich layers (Blanpain and Fuchs, 2006). If and how this heterogeneity and structural complexity influence the WE formation will be important to decipher. Indeed, during metamorphosis, amphibian skin goes through specialization and extensive changes (Schreiber and Brown, 2003; Seifert et al., 2012, 2019). In a parallel, unlike premetamorphic axolotl, postmetamorphic axolotl regenerates at a slower rate with a reduced regeneration ability (Monaghan et al., 2014), and a comparison between premetamorphic and postmetamorphic axolotl showed that the wound closure is slower in postmetamorphic animals (Seifert et al., 2012). These findings bring the possibility that the dynamics of epithelial movements may be a contributor to successful regeneration and pace of regeneration. On another note, it is well established that the immune system of regeneration-incompetent mammals operates differently compared to regeneration-competent species, and this difference could be impacting epithelial cells (Julier et al., 2017; Bolaños-Castro et al., 2021). Particularly, sustained inflammation does not interfere with the wound closure and therefore the WE formation (Godwin et al., 2013; Mescher et al., 2013; Cook and Seifert, 2016). By contrast, sustained inflammation is associated with failure to form an AEC in *Xenopus* tail regeneration and the axolotl limb regeneration (Godwin et al., 2013; Aztekin et al., 2020). However, it remains unclear which direct and indirect immune system-mediated mechanisms impact AEC formation. Further cross-species or animal state comparisons and mammalian epidermal biology studies can guide research to elucidate WE and AEC formation.

Single-cell methods have been providing new insights on cell types mediating regeneration and divergent features of the WE and the AEC. Further cross-species systematic investigations on these tissues, cell types, and their dynamic behaviors will reveal evolutionarily conserved genetic programs of epithelial signaling centers and their association with appendage growth and regeneration.

## Author Contributions

CA wrote the manuscript.

## Conflict of Interest

The author declares that the research was conducted in the absence of any commercial or financial relationships that could be construed as a potential conflict of interest.

## Publisher’s Note

All claims expressed in this article are solely those of the authors and do not necessarily represent those of their affiliated organizations, or those of the publisher, the editors and the reviewers. Any product that may be evaluated in this article, or claim that may be made by its manufacturer, is not guaranteed or endorsed by the publisher.

## References

[B1] Akimenko M. A. Johnson S. L. Westerfield M. Ekker M. (1995). Differential induction of four msx homeobox genes during fin development and regeneration in zebrafish. *Development* 121 347–357. 10.1242/dev.121.2.347 7768177

[B2] Aoki K. Kondo Y. Naoki H. Hiratsuka T. Itoh R. E. Matsuda M. (2017). Propagating wave of ERK activation orients collective cell migration. *Dev. Cell* 43 305–317.e5. 10.1016/j.devcel.2017.10.016 29112851

[B3] Aragona M. Dekoninck S. Rulands S. Lenglez S. Mascré G. Simons B. D. (2017). Defining stem cell dynamics and migration during wound healing in mouse skin epidermis. *Nat. Commun.* 8:14684. 10.1038/ncomms14684 28248284 PMC5339881

[B4] Arendt D. Musser J. M. Baker C. V. H. Bergman A. Cepko C. Erwin D. H. (2016). The origin and evolution of cell types. *Nat. Rev. Genet.* 17 744–757. 27818507 10.1038/nrg.2016.127

[B5] Aztekin C. (2021). Appendage regeneration is context dependent at the cellular level. *Open Biol.* 11:210126. 10.1098/rsob.210126 34315276 PMC8316798

[B6] Aztekin C. Hiscock T. W. Butler R. Andino F. D. J. Robert J. Gurdon J. B. (2020). The myeloid lineage is required for the emergence of a regeneration-permissive environment following Xenopus tail amputation. *Development* 147:dev185496. 10.1242/dev.185496 31988186 PMC7033733

[B7] Aztekin C. Hiscock T. W. Gurdon J. Jullien J. Marioni J. Simons B. D. (2021). Secreted inhibitors drive the loss of regeneration competence in Xenopus limbs. *Development* 148:dev199158. 10.1242/dev.199158 34105722 PMC8217717

[B8] Aztekin C. Hiscock T. W. Marioni J. C. Gurdon J. B. Simons B. D. Jullien J. (2019). Identification of a regeneration-organizing cell in the Xenopus tail. *Science* 364 653–658. 10.1126/science.aav9996 31097661 PMC6986927

[B9] Beauchemin M. Savard P. (1992). Two distal-less related homeobox-containing genes expressed in regeneration blastemas of the newt. *Dev. Biol.* 154 55–65. 10.1016/0012-1606(92)90047-k 1358728

[B10] Beck C. W. Christen B. Slack J. M. W. (2003). Molecular pathways needed for regeneration of spinal cord and muscle in a vertebrate. *Dev. Cell* 5 429–439. 10.1016/s1534-5807(03)00233-8 12967562

[B11] Beck C. W. Christen B. Barker D. Slack J. M. W. (2006). Temporal requirement for bone morphogenetic proteins in regeneration of the tail and limb of Xenopus tadpoles. *Mech. Dev.* 123 674–688. 10.1016/j.mod.2006.07.001 16938438

[B12] Beck C. W. Izpisúa Belmonte J. C. Christen B. (2009). Beyond early development: xenopus as an emerging model for the study of regenerative mechanisms. *Dev. Dyn.* 238 1226–1248. 10.1002/dvdy.21890 19280606

[B13] Blanpain C. Fuchs E. (2006). Epidermal stem cells of the skin. *Annu. Rev. Cell Dev. Biol.* 22 339–373. 16824012 10.1146/annurev.cellbio.22.010305.104357 PMC2405915

[B14] Boilly B. Cavanaugh K. P. Thomas D. Hondermarck H. Bryant S. V. Bradshaw R. A. (1991). Acidic fibroblast growth factor is present in regenerating limb blastemas of axolotls and binds specifically to blastema tissues. *Dev. Biol.* 145 302–310. 10.1016/0012-1606(91)90128-p 2040374

[B15] Bolaños-Castro L. A. Walters H. E. Vázquez R. O. G. Yun M. H. (2021). Immunity in salamander regeneration: where are we standing and where are we headed? *Dev. Dyn.* 250 753–767. 10.1002/dvdy.251 32924213 PMC8363947

[B16] Bryant D. M. Sousounis K. Farkas J. E. Bryant S. Thao N. Guzikowski A. R. (2017a). Repeated removal of developing limb buds permanently reduces appendage size in the highly-regenerative axolotl. *Dev. Biol.* 424 1–9. 10.1016/j.ydbio.2017.02.013 28235582 PMC5707178

[B17] Bryant D. M. Sousounis K. Payzin-Dogru D. Bryant S. Sandoval A. G. W. Martinez Fernandez J. (2017b). Identification of regenerative roadblocks via repeat deployment of limb regeneration in axolotls. *NPJ Regen. Med.* 2:30. 10.1038/s41536-017-0034-z 29302364 PMC5677943

[B18] Campbell L. J. Crews C. M. (2008). Wound epidermis formation and function in urodele amphibian limb regeneration. *Cell Mol. Life Sci.* 65 73–79. 10.1007/s00018-007-7433-z 18030417 PMC11131783

[B19] Cannata S. M. Bernardini S. Di Berardino R. Filoni S. (1992). Nerve-independent DNA synthesis and mitosis in regenerating hindlimbs of larval Xenopus laevis. *Roux’s Arch. Dev. Biol.* 201 128–133. 10.1007/BF00188710 28305578

[B20] Carlson M. R. Bryant S. V. Gardiner D. M. (1998). Expression of Msx-2 during development, regeneration, and wound healing in axolotl limbs. *J. Exp. Zool.* 282 715–723. 10.1002/(sici)1097-010x(19981215)282:6&lt;715::aid-jez7&gt;3.0.co;2-f 9846383

[B21] Castilla M. Tassava R. A. (1992). Extraction of the WE3 antigen and comparison of reactivities of mAbs WE3 and WE4 in adult newt regenerate epithelium and body tissues. *Monogr. Dev. Biol.* 23 116–130. 1614428

[B22] Choi H. M. T. Schwarzkopf M. Fornace M. E. Acharya A. Artavanis G. Stegmaier J. (2018). Third-generation in situ hybridization chain reaction: multiplexed, quantitative, sensitive, versatile, robust. *Development* 145:dev165753. 10.1242/dev.165753 29945988 PMC6031405

[B23] Christen B. Slack J. M. W. (1997). FGF-8Is associated with anteroposterior patterning and limb regeneration inXenopus. *Dev. Biol.* 192 455–466. 10.1006/dbio.1997.8732 9441681

[B24] Christensen R. N. Tassava R. A. (2000). Apical epithelial cap morphology and fibronectin gene expression in regenerating axolotl limbs. *Dev. Dyn.* 217 216–224. 10.1002/(SICI)1097-0177(200002)217:2&lt;216::AID-DVDY8&gt;3.0.CO;2-8 10706145

[B25] Christensen R. N. Weinstein M. Tassava R. A. (2002). Expression of fibroblast growth factors 4, 8, and 10 in limbs, flanks, and blastemas of Ambystoma. *Dev. Dyn.* 223 193–203. 10.1002/dvdy.10049 11836784

[B26] Cook A. B. Seifert A. W. (2016). Beryllium nitrate inhibits fibroblast migration to disrupt epimorphic regeneration. *Development* 143 3491–3505. 10.1242/dev.134882 27578793

[B27] Dawson L. A. Schanes P. P. Marrero L. Jordan K. Brunauer R. Zimmel K. N. (2021). Proximal digit tip amputation initiates simultaneous blastema and transient fibrosis formation and results in partial regeneration. *Wound Repair Regen.* 29 196–205. 10.1111/wrr.12856 32815252

[B28] Del Rio-Tsonis K. Washabaugh C. H. Tsonis P. A. (1992). The mutant axolotl short toes exhibits impaired limb regeneration and abnormal basement membrane formation. *Proc. Natl. Acad. Sci. USA* 89 5502–5506. 10.1073/pnas.89.12.5502 1608961 PMC49320

[B29] Dent J. N. (1962). Limb regeneration in larvae and metamorphosing individuals of the South African clawed toad. *J. Morphol.* 110 61–77. 10.1002/jmor.1051100105 13885494

[B30] Dolan C. P. Dawson L. A. Muneoka K. (2018). Digit tip regeneration: merging regeneration biology with regenerative medicine. *Stem Cells Transl. Med.* 7 262–270. 10.1002/sctm.17-0236 29405625 PMC5827737

[B31] Endo T. Tamura K. Ide H. (2000). Analysis of gene expressions during xenopus forelimb regeneration. *Dev. Biol.* 220 296–306. 10753517 10.1006/dbio.2000.9641

[B32] Fernandez-Teran M. Ros M. A. (2008). The apical ectodermal ridge: morphological aspects and signaling pathways. *Int. J. Dev. Biol.* 52 857–871. 10.1387/ijdb.072416mf 18956316

[B33] Francis-West P. H. Antoni L. Anakwe K. (2003). Regulation of myogenic differentiation in the developing limb bud. *J. Anat.* 202 69–81. 12587922 10.1046/j.1469-7580.2003.00136.x PMC1571055

[B34] Gagliardi P. A. Dobrzyński M. Jacques M.-A. Dessauges C. Ender P. Blum Y. (2021). Collective ERK/Akt activity waves orchestrate epithelial homeostasis by driving apoptosis-induced survival. *Dev. Cell* 56 1712–1726.e6. 10.1016/j.devcel.2021.05.007 34081908

[B35] Gerber T. Murawala P. Knapp D. Masselink W. Schuez M. Hermann S. (2018). Single-cell analysis uncovers convergence of cell identities during axolotl limb regeneration. *Science* 362:eaaq0681. 10.1126/science.aaq0681 30262634 PMC6669047

[B36] Ghosh S. Roy S. Séguin C. Bryant S. V. Gardiner D. M. (2008). Analysis of the expression and function of Wnt-5a and Wnt-5b in developing and regenerating axolotl (*Ambystoma mexicanum*) limbs. *Dev. Growth Differ.* 50 289–297. 10.1111/j.1440-169X.2008.01000.x 18336582

[B37] Godwin J. W. Pinto A. R. Rosenthal N. A. (2013). Macrophages are required for adult salamander limb regeneration. *Proc. Natl. Acad. Sci. U.S.A.* 110 9415–9420. 23690624 10.1073/pnas.1300290110 PMC3677454

[B38] Goldhamer D. J. Tomlinson B. L. Tassava R. A. (1989). A developmentally regulated wound epithelial antigen of the newt limb regenerate is also present in a variety of secretory/transport cell types. *Dev. Biol.* 135 392–404. 10.1016/0012-1606(89)90188-7 2506090

[B39] Goss R. J. (1956). Regenerative inhibition following limb amputation and immediate insertion into the body cavity. *Anat. Rec.* 126 15–27. 10.1002/ar.1091260103 13362966

[B40] Gumpel-Pinot M. Ede D. A. Flint O. P. (1984). Myogenic cell movement in the developing avian limb bud in presence and absence of the apical ectodermal ridge (AER). *Development* 80 105–125. 6747521

[B41] Han M.-J. An J.-Y. Kim W.-S. (2001). Expression patterns of Fgf-8 during development and limb regeneration of the axolotl. *Dev. Dyn.* 220 40–48. 10.1002/1097-0177(2000)9999:9999&lt;::AID-DVDY1085&gt;3.0.CO;2-8 11146506

[B42] Hay E. D. Fischman D. A. (1961). Origin of the blastema in regenerating limbs of the newt Triturus viridescens: an autoradiographic study using tritiated thymidine to follow cell proliferation and migration. *Dev. Biol.* 3 26–59. 10.1016/0012-1606(61)90009-4 13712434

[B43] Hino N. Rossetti L. Marín-Llauradó A. Aoki K. Trepat X. Matsuda M. (2020). ERK-mediated mechanochemical waves direct collective cell polarization. *Dev. Cell* 53 646–660.e8. 10.1016/j.devcel.2020.05.011 32497487

[B44] Joven A. Elewa A. Simon A. (2019). Model systems for regeneration: salamanders. *Development* 146:dev167700. 31332037 10.1242/dev.167700 PMC6679358

[B45] Julier Z. Park A. J. Briquez P. S. Martino M. M. (2017). Promoting tissue regeneration by modulating the immune system. *Acta Biomater.* 53 13–28. 28119112 10.1016/j.actbio.2017.01.056

[B46] Kawakami Y. Capdevila J. Büscher D. Itoh T. Esteban C. R. Belmonte J. C. I. (2001). WNT signals control FGF-dependent limb initiation and AER induction in the chick embryo. *Cell* 104 891–900. 10.1016/s0092-8674(01)00285-9 11290326

[B47] Kawakami Y. Rodriguez Esteban C. Raya M. Kawakami H. Martí M. Dubova I. (2006). Wnt/β-catenin signaling regulates vertebrate limb regeneration. *Genes Dev.* 20 3232–3237. 10.1002/1097-0177(2000)9999:9999&lt;::aid-dvdy1045&gt;3.3.co;2-3 17114576 PMC1686599

[B48] Kelley R. O. Fallon J. F. (1976). Ultrastructural analysis of the apical ectodermal ridge during vertebrate limb morphogenesis: i. the human forelimb with special reference to gap junctions. *Dev. Biol.* 51 241–256. 10.1016/0012-1606(76)90141-x 955259

[B49] Kishi J. Y. Lapan S. W. Beliveau B. J. West E. R. Zhu A. Sasaki H. M. (2019). SABER amplifies FISH: enhanced multiplexed imaging of RNA and DNA in cells and tissues. *Nat. Methods* 16 533–544. 10.1038/s41592-019-0404-0 31110282 PMC6544483

[B50] Knapp D. Schulz H. Rascon C. A. Volkmer M. Scholz J. Nacu E. (2013). Comparative transcriptional profiling of the axolotl limb identifies a tripartite regeneration-specific gene program. *PLoS One* 8:e61352. 10.1371/journal.pone.0061352 23658691 PMC3641036

[B51] Lash J. W. (1955). Studies on wound closure in urodeles. *J. Exp. Zool.* 128 13–28. 10.1002/ar.b.20082 16308860

[B52] Lehoczky J. A. Robert B. Tabin C. J. (2011). Mouse digit tip regeneration is mediated by fate-restricted progenitor cells. *Proc. Natl. Acad. Sci. U.S.A.* 108 20609–20614. 10.1073/pnas.1118017108 22143790 PMC3251149

[B53] Leigh N. D. Dunlap G. S. Johnson K. Mariano R. Oshiro R. Wong A. Y. (2018). Transcriptomic landscape of the blastema niche in regenerating adult axolotl limbs at single-cell resolution. *Nat. Commun.* 9:5153. 10.1038/s41467-018-07604-0 30514844 PMC6279788

[B54] Li C. Yang F. Li G. Gao X. Xing X. Wei H. (2007). Antler regeneration: a dependent process of stem tissue primed via interaction with its enveloping skin. *J. Exp. Zool. Part A Ecol. Genet. Physiol.* 307A 95–105. 10.1002/jez.a.352 17177282

[B55] Li C. Zhao H. Liu Z. McMahon C. (2014). Deer antler – a novel model for studying organ regeneration in mammals. *Int. J. Biochem. Cell Biol.* 56 111–122. 10.1016/j.biocel.2014.07.007 25046387

[B56] Li H. Wei X. Zhou L. Zhang W. Wang C. Guo Y. (2020). Dynamic cell transition and immune response landscapes of axolotl limb regeneration revealed by single-cell analysis. *Protein Cell* 12 57–66. 10.1007/s13238-020-00763-1 32748350 PMC7815851

[B57] Lin G. Slack J. M. W. (2008). Requirement for Wnt and FGF signaling in Xenopus tadpole tail regeneration. *Dev. Biol.* 316 323–335. 18329638 10.1016/j.ydbio.2008.01.032

[B58] Mariani F. V. Ahn C. P. Martin G. R. (2008). Genetic evidence that FGFs have an instructive role in limb proximal–distal patterning. *Nature* 453 401–405. 10.1038/nature06876 18449196 PMC2631409

[B59] Mateus R. Pereira T. Sousa S. Lima J. E. Pascoal S. Saúde L. (2012). In vivo cell and tissue dynamics underlying zebrafish fin fold regeneration. *PLoS One* 7:e51766. 10.1371/journal.pone.0051766 23284763 PMC3527495

[B60] McKinley K. L. Castillo-Azofeifa D. Klein O. D. (2020). Tools and concepts for interrogating and defining cellular identity. *Cell Stem Cell* 26 632–656. 10.1016/j.stem.2020.03.015 32386555 PMC7250495

[B61] McQueen C. Towers M. (2020). Establishing the pattern of the vertebrate limb. *Development* 147:dev177956. 32917670 10.1242/dev.177956

[B62] McQueeney K. Soufer R. Dealy C. N. (2002). β-Catenin-dependent Wnt signaling in apical ectodermal ridge induction and FGF8 expression in normal and limbless mutant chick limbs. *Dev. Growth Differ.* 44 315–325. 10.1046/j.1440-169x.2002.00647.x 12175366

[B63] Mescher A. L. (1976). Effects on adult newt limb regeneration of partial and complete skin flaps over the amputation surface. *J. Exp. Zool.* 195 117–127. 10.1002/jez.1401950111 1255117

[B64] Mescher A. L. Neff A. W. King M. W. (2013). Changes in the inflammatory response to injury and its resolution during the loss of regenerative capacity in developing xenopus limbs. *PLoS One* 8:e80477. 10.1371/journal.pone.0080477 24278286 PMC3835323

[B65] Monaghan J. R. Athippozhy A. Seifert A. W. Putta S. Stromberg A. J. Maden M. (2012). Gene expression patterns specific to the regenerating limb of the Mexican axolotl. *Biol. Open* 1 937–948. 10.1242/bio.20121594 23213371 PMC3507169

[B66] Monaghan J. R. Stier A. C. Michonneau F. Smith M. D. Pasch B. Maden M. (2014). Experimentally induced metamorphosis in axolotls reduces regenerative rate and fidelity. *Regeneration* 1 2–14. 10.1002/reg2.8 27499857 PMC4895291

[B67] Moriyasu M. Makanae A. Satoh A. (2012). Spatiotemporal regulation of keratin 5 and 17 in the axolotl limb. *Dev. Dyn.* 241 1616–1624. 10.1002/dvdy.23839 22836940

[B68] Morosow J. (1938). The inhibition and restoration of the regeneration process of the extremities in the axolotl. *C. R. Acad. Sci. URSS* 20 207–210.

[B69] Mullen L. M. Bryant S. V. Torok M. A. Blumberg B. Gardiner D. M. (1996). Nerve dependency of regeneration: the role of Distal-less and FGF signaling in amphibian limb regeneration. *Development* 122 3487–3497. 10.1242/dev.122.11.3487 8951064

[B70] Nacu E. Gromberg E. Oliveira C. R. Drechsel D. Tanaka E. M. (2016). FGF8 and SHH substitute for anterior–posterior tissue interactions to induce limb regeneration. *Nature* 533 407–410. 10.1038/nature17972 27120163

[B71] Nakamura H. Yasuda M. (1979). An electron microscopic study of periderm cell development in mouse limb buds. *Anat. Embryol.* 157 121–132. 10.1007/bf00305153 517761

[B72] Neufeld D. A. Day F. A. Settles H. E. (1996). Stabilizing role of the basement membrane and dermal fibers during newt limb regeneration. *Anat. Rec.* 245 122–127. 10.1002/(SICI)1097-0185(199605)245:1&lt;122::AID-AR17&gt;3.0.CO;2-R 8731048

[B73] Ohuchi H. Nakagawa T. Itoh N. Noji S. (1999). FGF10 can induce Fgf8 expression concomitantly with En1 and R-fng expression in chick limb ectoderm, independent of its dorsoventral specification. *Dev. Growth Differ.* 41 665–673. 10.1046/j.1440-169x.1999.00466.x 10646796

[B74] Ohuchi H. Nakagawa T. Yamamoto A. Araga A. Ohata T. Ishimaru Y. (1997). The mesenchymal factor, FGF10, initiates and maintains the outgrowth of the chick limb bud through interaction with FGF8, an apical ectodermal factor. *Development* 124 2235–2244. 10.1242/dev.124.11.2235 9187149

[B75] Okumura A. Hayashi T. Ebisawa M. Yoshimura M. Sasagawa Y. Nikaido I. (2019). Cell type-specific transcriptome analysis unveils secreted signaling molecule genes expressed in apical epithelial cap during appendage regeneration. *Dev. Growth Differ.* 61 447–456. 10.1111/dgd.12635 31713234

[B76] Pearl E. J. Barker D. Day R. C. Beck C. W. (2008). Identification of genes associated with regenerative success of Xenopus laevis hindlimbs. *BMC Dev. Biol.* 8:66. 10.1186/1471-213X-8-66 18570684 PMC2483965

[B77] Pfefferli C. Jaźwińska A. (2015). The art of fin regeneration in zebrafish. *Regeneration* 2 72–83. 10.1002/reg2.33 27499869 PMC4895310

[B78] Pickering J. Rich C. A. Stainton H. Aceituno C. Chinnaiya K. Saiz-Lopez P. (2018). An intrinsic cell cycle timer terminates limb bud outgrowth. *ELife* 7:e37429. 10.7554/eLife.37429 30175958 PMC6143340

[B79] Poss K. D. Shen J. Keating M. T. (2000). Induction of lef1 during zebrafish fin regeneration. *Dev. Dyn.* 219 282–286. 11002347 10.1002/1097-0177(2000)9999:9999<::aid-dvdy1045>3.3.co;2-3

[B80] Purushothaman S. Elewa A. Seifert A. W. (2019). Fgf-signaling is compartmentalized within the mesenchyme and controls proliferation during salamander limb development. *ELife* 8:e48507. 31538936 10.7554/eLife.48507 PMC6754229

[B81] Qin T. Fan C.-M. Wang T.-Z. Sun H. Zhao Y.-Y. Yan R.-J. (2020). Single-cell RNA-seq reveals novel mitochondria-related musculoskeletal cell populations during adult axolotl limb regeneration process. *Cell Death Differ.* 28 111–1125. 10.1038/s41418-020-00640-8 33116295 PMC7937690

[B82] Repesh L. A. Oberpriller J. C. (1978). Scanning electron microscopy of epidermal cell migration in wound healing during limb regeneration in the adult newt, Notophthalmus viridescens. *Am. J. Anat.* 151 539–555. 645617 10.1002/aja.1001510408

[B83] Rodgers A. K. Smith J. J. Voss S. R. (2020). Identification of immune and non-immune cells in regenerating axolotl limbs by single-cell sequencing. *Exp. Cell Res.* 394:112149. 10.1016/j.yexcr.2020.112149 32562784 PMC7483677

[B84] Satoh A. Graham G. M. C. Bryant S. V. Gardiner D. M. (2008). Neurotrophic regulation of epidermal dedifferentiation during wound healing and limb regeneration in the axolotl (*Ambystoma mexicanum*). *Dev. Biol.* 319 321–335. 10.1016/j.ydbio.2008.04.030 18533144

[B85] Satoh A. Ide H. Tamura K. (2005). Muscle formation in regenerating Xenopus froglet limb. *Dev. Dyn.* 233 337–346. 10.1002/dvdy.20349 15768391

[B86] Satoh A. Mitogawa K. Saito N. Suzuki M. Suzuki K. T. Ochi H. (2017). Reactivation of larval keratin gene (krt62.L) in blastema epithelium during Xenopus froglet limb regeneration. *Dev. Biol.* 432 265–272. 10.1016/j.ydbio.2017.10.015 29079423

[B87] Saunders J. W. (1948). The proximo-distal sequence of origin of the parts of the chick wing and the role of the ectoderm. *J. Exp. Zool.* 108 363–403. 10.1002/jez.1401080304 18882505

[B88] Scherz P. J. Harfe B. D. McMahon A. P. Tabin C. J. (2004). The limb bud Shh-Fgf feedback loop is terminated by expansion of former ZPA cells. *Science* 305 396–399. 10.1126/science.1096966 15256670

[B89] Schreiber A. M. Brown D. D. (2003). Tadpole skin dies autonomously in response to thyroid hormone at metamorphosis. *Proc. Natl. Acad. Sci. U.S.A.* 100 1769–1774. 10.1073/pnas.252774999 12560472 PMC149908

[B90] Scott C. A. Carney T. J. Amaya E. (2021). Aerobic glycolysis is important for zebrafish larval wound closure and tail regeneration. *BioRxiv* Available Online at: 10.1101/2021.04.23.441208 (accessed September 2, 2021). PMC9828577 36148505

[B91] Seifert A. W. Cook A. B. Shaw D. (2019). Inhibiting fibroblast aggregation in skin wounds unlocks developmental pathway to regeneration. *Dev. Biol.* 455 60–72. 10.1016/j.ydbio.2019.07.001 31279726

[B92] Seifert A. W. Monaghan J. R. Voss S. R. Maden M. (2012). Skin regeneration in adult axolotls: a blueprint for scar-free healing in vertebrates. *PLoS One* 7:e32875. 10.1371/journal.pone.0032875 22485136 PMC3317654

[B93] Shibata E. Yokota Y. Horita N. Kudo A. Abe G. Kawakami K. (2016). Fgf signalling controls diverse aspects of fin regeneration. *Development* 143 2920–2929. 10.1242/dev.140699 27402707

[B94] Shimizu-Nishikawa K. Tazawa I. Uchiyama K. Yoshizato K. (1999). Expression of helix-loop-helix type negative regulators of differentiation during limb regeneration in urodeles and anurans. *Dev. Growth Differ.* 41 731–743. 10.1046/j.1440-169x.1999.00477.x 10646803

[B95] Shimokawa T. Yasutaka S. Kominami R. Shinohara H. (2012). Wound epithelium function in axolotl limb regeneration. *Okajimas Folia Anat. Jpn.* 89 75–81. 10.2535/ofaj.89.75 23429052

[B96] Simkin J. Sammarco M. C. Dawson L. A. Tucker C. Taylor L. J. Van Meter K. (2015). Epidermal closure regulates histolysis during mammalian (Mus) digit regeneration. *Regeneration* 2 106–119. 10.1002/reg2.34 27499872 PMC4895321

[B97] Stocum D. L. (2019). Nerves and proliferation of progenitor cells in limb regeneration. *Dev. Neurobiol.* 79 468–478. 10.1002/dneu.22643 30303627

[B98] Stopper G. F. Wagner G. P. (2005). Of chicken wings and frog legs: a smorgasbord of evolutionary variation in mechanisms of tetrapod limb development. *Dev. Biol.* 288 21–39. 16246321 10.1016/j.ydbio.2005.09.010

[B99] Storer M. Mas A. Robert-Moreno A. Pecoraro M. Ortells M. C. Di Giacomo V. (2013). Senescence is a developmental mechanism that contributes to embryonic growth and patterning. *Cell* 155 1119–1130. 10.1016/j.cell.2013.10.041 24238961

[B100] Sugiura T. Wang H. Barsacchi R. Simon A. Tanaka E. M. (2016). MARCKS-like protein is an initiating molecule in axolotl appendage regeneration. *Nature* 531 237–240. 10.1038/nature16974 26934225 PMC4795554

[B101] Suzuki M. Satoh A. Ide H. Tamura K. (2005). Nerve-dependent and -independent events in blastema formation during Xenopus froglet limb regeneration. *Dev. Biol.* 286 361–375. 10.1016/j.ydbio.2005.08.021 16154125

[B102] Tank P. W. Carlson B. M. Connelly T. G. (1977). A scanning electron microscopic comparison of the development of embryonic and regenerating limbs in the axolotl. *J. Exp. Zool.* 201 417–429. 10.1002/jez.1402010308 908913

[B103] Tassava R. A. Acton R. D. (1989). Distribution of a wound epithelium antigen in embryonic tissues of newts and salamanders. *Ohio J. Sci.* 89 12–15.

[B104] Tassava R. A. Garling D. J. (1979). Regenerative responses in larval axolotl limbs with skin grafts over the amputation surface. *J. Exp. Zool.* 208 97–109. 10.1002/jez.1402080111 381569

[B105] Tassava R. A. Loyd R. M. (1977). Injury requirement for initiation of regeneration of newt limbs which have whole skin grafts. *Nature* 268 49–50. 10.1038/268049a0 329140

[B106] Tassava R. A. Mescher A. L. (1975). The roles of injury, nerves, and the wound epidermis during the initiation of amphibian limb regeneration. *Differentiation* 4 23–24. 10.1111/j.1432-0436.1975.tb01439.x 1205030

[B107] Tassava R. A. Olsen C. L. (1982). Higher vertebrates do not regenerate digits and legs because the wound epidermis is not functional: a hypothesis. *Differentiation* 22 151–155. 10.1111/j.1432-0436.1982.tb01242.x 7173524

[B108] Tassava R. A. Castilla M. Arsanto J. P. Thouveny Y. (1993). The wound epithelium of regenerating limbs of Pleurodeles waltl and Notophthalmus viridescens: studies with mAbs WE3 and WE4, phalloidin, and DNase 1. *J. Exp. Zool.* 267 180–187. 10.1002/jez.1402670211 8409899

[B109] Tassava R. A. Johnson-Wint B. Gross J. (1986). Regenerate epithelium and skin glands of the adult newt react to the same monoclonal antibody. *J. Exp. Zool.* 239 229–240. 10.1002/jez.1402390210 3528384

[B110] Thornton C. S. (1957). The effect of apical cap removal on limb regeneration in Amblystoma larvae. *J. Exp. Zool.* 134 357–381. 10.1002/jez.1401340209 13428959

[B111] Thornton C. S. (1958). The inhibition of limb regeneration in urodele larvae by localized irradiation with ultraviolet light. *J. Exp. Zool.* 137 153–179. 10.1002/jez.1401370108 13563789

[B112] Thornton C. S. (1960). Influence of an eccentric epidermal cap on limb regeneration in Amblystoma larvae. *Dev. Biol.* 2 551–569. 10.1016/0012-1606(60)90054-3 13776717

[B113] Thornton C. S. Thornton M. T. (1965). The regeneration of accessory limb parts following epidermal cap transplantation in urodeles. *Experientia* 21 146–148. 10.1007/BF02141984 5318741

[B114] Trapnell C. (2015). Defining cell types and states with single-cell genomics. *Genome Res.* 25 1491–1498. 10.1101/gr.190595.115 26430159 PMC4579334

[B115] Tsai S. L. Baselga-Garriga C. Melton D. A. (2019). Blastemal progenitors modulate immune signaling during early limb regeneration. *Development* 146:dev169128. 10.1242/dev.169128 30602532

[B116] Tsai S. L. Baselga-Garriga C. Melton D. A. (2020). Midkine is a dual regulator of wound epidermis development and inflammation during the initiation of limb regeneration. *ELife* 9:e50765. 10.7554/eLife.50765 31934849 PMC6959999

[B117] Tschumi P. A. (1957). The growth of the hindlimb bud of Xenopus laevis and its dependence upon the epidermis. *J. Anat.* 91 (Pt 2) 149–173. 13416124 PMC1244934

[B118] Verheyden J. M. Sun X. (2008). An Fgf/gremlin inhibitory feedback loop triggers termination of limb bud outgrowth. *Nature* 454 638–641. 10.1038/nature07085 18594511 PMC2840222

[B119] Vinarsky V. Atkinson D. L. Stevenson T. J. Keating M. T. Odelberg S. J. (2005). Normal newt limb regeneration requires matrix metalloproteinase function. *Dev. Biol.* 279 86–98. 10.1016/j.ydbio.2004.12.003 15708560

[B120] Vincent E. Villiard E. Sader F. Dhakal S. Kwok B. H. Roy S. (2020). BMP signaling is essential for sustaining proximo-distal progression in regenerating axolotl limbs. *Development* 147:dev170829. 10.1242/dev.170829 32665245

[B121] Vitulo N. Dalla Valle L. Skobo T. Valle G. Alibardi L. (2017). Transcriptome analysis of the regenerating tail vs. the scarring limb in lizard reveals pathways leading to successful vs. unsuccessful organ regeneration in amniotes: tail and limb transcriptome in regenerating lizard. *Dev. Dyn.* 246 116–134. 10.1002/dvdy.24474 27870483

[B122] Wang Y.-H. Beck C. W. (2014). Distal expression of sprouty (spry) genes during Xenopus laevis limb development and regeneration. *Gene Expr. Patterns* 15 61–66. 10.1016/j.gep.2014.04.004 24823862

[B123] Watanabe A. Ohsugi K. Ide H. (1993). Formation of distal structures from stumps of chick wing buds at stages 24-25 following the grafting of quail tissue from X-irradiated distal limb buds. *J. Exp. Zool.* 267 447–453. 10.1002/jez.1402670410 8270896

[B124] Wei Y. Yang E. V. Klatt K. P. Tassava R. A. (1995). Monoclonal antibody MT2 identifies the urodele alpha 1 chain of type XII collagen, a developmentally regulated extracellular matrix protein in regenerating newt limbs. *Dev. Biol.* 168 503–513. 10.1006/dbio.1995.1098 7729585

[B125] Whited J. L. Lehoczky J. A. Austin C. A. Tabin C. J. (2011). Dynamic expression of two thrombospondins during axolotl limb regeneration. *Dev. Dyn.* 240 1249–1258. 10.1002/dvdy.22548 21360624 PMC3081376

[B126] Yang E. V. Gardiner D. M. Carlson M. R. Nugas C. A. Bryant S. V. (1999). Expression of Mmp-9 and related matrix metalloproteinase genes during axolotl limb regeneration. *Dev. Dyn.* 216 2–9. 10.1002/(SICI)1097-0177(199909)216:1&lt;2::AID-DVDY2&gt;3.0.CO;2-P 10474160

[B127] Yokoyama H. Ide H. Tamura K. (2001). FGF-10 stimulates limb regeneration ability in Xenopus laevis. *Dev. Biol.* 233 72–79. 10.1006/dbio.2001.0180 11319858

[B128] Yonei-Tamura S. Endo T. Yajima H. Ohuchi H. Ide H. Tamura K. (1999). FGF7 and FGF10 directly induce the apical ectodermal ridge in chick embryos. *Dev. Biol.* 211 133–143. 10.1006/dbio.1999.9290 10373311

[B129] Yoshida K. Kawakami K. Abe G. Tamura K. (2020). Zebrafish can regenerate endoskeleton in larval pectoral fin but the regenerative ability declines. *Dev. Biol.* 463 110–123. 10.1016/j.ydbio.2020.04.010 32422142

